# Assessment of dopperfluxometric indices of maternal-fetal structures in pregnant ewes

**DOI:** 10.1590/1984-3143-AR2021-0002

**Published:** 2021-07-05

**Authors:** Victor José Correia Santos, Mariana Garcia Kako Rodriguez, Priscila Del Aguila da Silva, Renata Sitta Gomes Mariano, Augusto Ryonosuke Taira, Luciana Cristina Padilha-Nakaghi, Ricardo Andres Ramirez Uscategui, Marcus Antonio Rossi Feliciano, Maria Emilia Franco Oliveira, Paola Castro Moraes, Wilter Ricardo Russiano Vicente

**Affiliations:** 1 Departamento de Reprodução Animal, Faculdade de Ciências Agrárias e Veterinárias, Universidade Estadual Paulista “Júlio de Mesquita Filho”, Jaboticabal, SP, Brasil; 2 Instituto de Ciências Agrárias, Universidade Federal dos Vales do Jequitinhonha e Mucuri, Unaí, MG, Brasil; 3 Departamento de Clínica e Cirurgia de Grandes Animais, Universidade Federal de Santa Maria, Santa Maria, RS, Brasil; 4 Universidade Estadual Paulista “Júlio de Mesquita Filho”, Faculdade de Ciências Agrárias e Veterinárias, Departamento de Clínica e Cirurgia Veterinárias, Jaboticabal, SP, Brasil

**Keywords:** ultrasonography, Doppler, sheep, pregnancy

## Abstract

The aim of this study was to evaluate the blood flow of the uterine artery, fetal aorta and umbilical artery in the physiological pregnancy of sheep by means of pulsed Doppler throughout the gestational period. Thirty Santa Inês ewes weighing between 45.4±4.3 kg and aged 2 to 5 years were selected. The evaluations were carried out weekly from the 3^rd^ to the 21^st^ gestational week. Peak systolic velocity (PSV), end diastolic velocity (EDV) and resistance index (RI) were obtained. Analysis of variance was performed, and the minimum significant comparison of means was obtained by the BH test with adjusted *P*<0.05. The results were expressed as mean ± standard error. For the fetal aorta, there was an increase in the EDV values and a decrease in the PSV and RI throughout pregnancy. For the uterine artery, PSV and EDV did not present significant variation, whereas the RI showed a reduction in the last week. Increased EDV values were found for the umbilical artery throughout pregnancy. For the PSV there was no significant difference, as the RI was reduced at the end of pregnancy. The results obtained are expected to contribute to a more complete understanding of the hemodynamic changes resulting from pregnancy.

## Introduction

It is essential that the embryo/fetus and the structures related to pregnancy receive an adequate blood supply and, for this end, several variations (Sarafana et al., 2007), occur involving the hemodynamics of the circulatory system and are characterized mainly by the increase in cardiac output and decreasing of the systemic vascular resistance (Hameed and Sklansky, 2007), assuring uterine perfusion and fetal development (Eghbali et al., 2005; Blanco et al., 2008).

Doppler ultrasonography has been used successfully in the evaluation of parameters related to blood flow and vascularization on different tissues of the animals' reproductive system (Feliciano et al., 2013) and its applicability has been tested and validated in small ruminants as a predictor method of pregnancy, by assessing ovarian blood flow (Bragança et al., 2016) with 100% accuracy at 17 days of pregnancy, and in studies concerning gestational vascularization (Acharya et al., 2007; Elmetwally and Bollwein, 2016; Beltrame et al., 2017).

According to Khong (2004) impaired placental perfusion, due to inadequate trophoblastic invasion of the spiral arteries, is associated with pre-eclampsia and restricted intrauterine fetal growth. For this reason, Acharya et al. (2007) emphasize that Doppler can be used to identify pregnancies at risk of developing such complications. These authors measured the volume of blood flow in the uterine artery during the pregnancy of sheep and concluded that the absolute velocities of this flow reflect the volume of uteroplacental blood flow in pregnant sheep.

Elmetwally and Bollwein (2016) evaluated the behavior of blood flow in the uterine artery ipsilateral to the pregnant horn to assess the applicability of Doppler technology in the gestational evaluation of sheep. They found that during pregnancy, there is a reduction in the diameter of the uterine artery with a positive correlation (*P*<0.05) between it and blood volume (r=0.62) and acceleration of blood flow and mean flow speed (r=0.32) respectively. The authors also highlight the potential for predicting gestational age and the possibility of mapping blood flow, with practical applicability in the obstetric routine.

In sheep there are few studies on the evaluation throughout the gestational period, the blood flow in the vessels involved in this process and it is known that hemodynamics in pregnancy is related to embryonic/fetal development, therefore, the objective was to obtain the dopplerfluxometric indices assessed throughout the gestational period in order to contribute to increase knowledge about the physiology of pregnancy in this species.

## Materials and Methods

This study was approved by the Ethics Committee on the Use of Animals (CEUA) of the Faculty of Agricultural and Veterinary Sciences of the Universidade Estadual Paulista, Jaboticabal, SP, Brazil (protocol nº11708-14). Thirty Santa Inês sheep, pluriparous, adult, healthy (aged between 2 and 5 years) and with average body weight of 45.4 ± 4.3kg were selected.

The animals belonged to the Department of Preventive Veterinary Medicine and Animal Reproduction, kept in pens, fed with corn silage, commercial feed and water *ad libitum*. Thirty days before starting the experimental period, the animals were conditioned to perform trichotomy of the abdominal region and ultrasound exams, to minimize the stress induced by the experimental procedures.

After that, the animals were submitted to the heat induction protocol as mentioned by Souza et al. (2007). After 24 hours, the females were allocated for a period of two days with a ram with proven reproductive efficiency. A mixture of soy oil and dye was applied to the animal's chest to identify mated females.

The day after identification of mating was considered the 1^st^ gestational day and on the 21^st^ day ultrasound evaluation was performed using the B-mode transrectally for the diagnosis of pregnancy, characterized by the visualization of the embryonic vesicle with the embryo inside and evaluation of fetal viability by identifying heartbeats. B-mode was also used to obtain the approximate location of the evaluated vessels, which was confirmed by color Doppler. If the pregnancy diagnosis was positive, weekly ultrasound assessments were started immediately, with the animals in a quadrupedal position by means of physical restraint.

The transrectal approach was used until the 8^th^ week of pregnancy since the uterus was located dorsally in this evaluation period, being closer to the probe, thus facilitating the acquisition of images. In addition, the sheep's abdomen was positioned on the operator's leg so that the distance between the uterus and the transducer was reduced. From the 9^th^ week, the transabdominal route (adapted from Elmetwally et al, 2016) was used, due to the discomfort with the positioning described previously and the fact that the uterus is located closer to the abdominal wall due to weight gain. It was necessary to perform abdominal trichotomy when the transabdominal route was used and with the advancement of pregnancy it was necessary to enlarge the trichotomized area due to the size of the fetuses that started to occupy a large part of the abdominal cavity.

The examinations were carried out with the MyLab ™ 30 VET equipment (Esaote S.p.A., Genova, Liguria, Italy) linear transducer (7.5MHz) through the rectum until the 8^th^ week of gestation. From the 9^th^ to the week before delivery, a linear transducer (7.5MHz) was used transabdominally.

After identifying the vessels by color Doppler, a sample volume (gate) had its size adjusted (2±4mm), depending on the diameter of the vessels, and was positioned in the central portion of them. Then, pulsed Doppler was activated to obtain the spectral tracing and vascular indices. A minimum of three waves was used in the assessment. After the correction of the insonation angle (maximum 60º) and with the tracing free of artifacts, the images were frozen and the wave morphology was analyzed, obtaining the peak systolic velocity (PSV), end diastolic velocity (EDV) and resistance index (RI = [PSV-EDV] / PSV). The indices were determined automatically (Feliciano et al., 2012).

The following vessels were evaluated weekly: uterine arteries (*n*=299), umbilical arteries (*n*=311) and fetal aorta (*n*=236).

Statistics were performed using the R software (R^®^ foundation for statistical computing, Austria) and according to the recommendations of Chen and Peace (2011). The analysis of variance was performed for repeated measures over time with the approximation of the Satterthwaite degrees of freedom. For this purpose, the lmerTest package (Kuznetsova et al., 2017) was used and the minimum significant comparison of the means was obtained by the BH test (Benjamini and Hochberg, 1995) being considered significant for the adjusted *P*<0.05 (*P* adj<0.05).

## Results

From the total of 30 ewes, 22 pregnancies were single, seven twins and one triplet. All pregnancies were at term, the births took place without the need for intervention and 39 lambs were born healthy.

The conditioning contributed for the animal’s acceptance of handling without causing great stress, which was key for the pregnancies to occur without complications. Transabdominal examination from the 9^th^ week onwards was fundamental due to the impossibility of continuing the evaluations transrectally.

Cross-sectional images of the fetus facilitated the evaluation of the fetal aorta, located below the spine and characterized as a circular structure with the presence of blood flow confirmed by color Doppler. It was also possible to locate it in the longitudinal section, however, the equipment often had trouble to form the spectral Doppler tracing.

The period (gestational weeks) during which it was possible to make the assessment on the proposed arteries was: fetal aorta (5 – 19); Uterine (3 – 21); Umbilical (4 – 21).

### Fetal aorta

**Figure 1:**
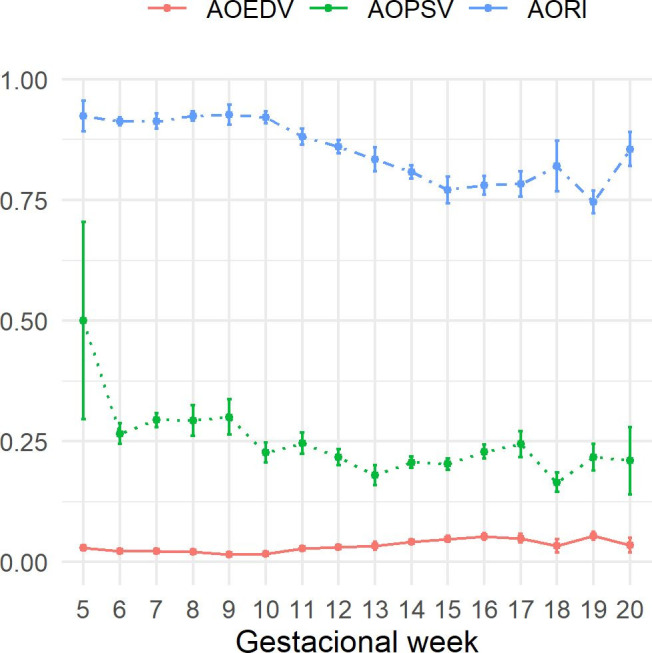
Graph of mean ± standard error of fetal aorta’s end diastolic velocity (cm/s), peak systolic velocity (cm/s) and resistance index, of Santa Inês sheep fetuses throughout pregnancy. Even though (*P*>0.005), peak systolic velocity and end diastolic velocity presented higher values at the end of pregnancy and resistance index was reduced throughout pregnancy.

For the fetal aorta, the end diastolic velocity presented lower values in the initial weeks, with greater values being observed in the last weeks of pregnancy. Peak systolic velocity with higher values obtained in the final weeks of pregnancy when compared to the initial weeks and there was a reduction in the resistance index values during pregnancy (Figure 1).

### Uterine artery

**Figure 2:**
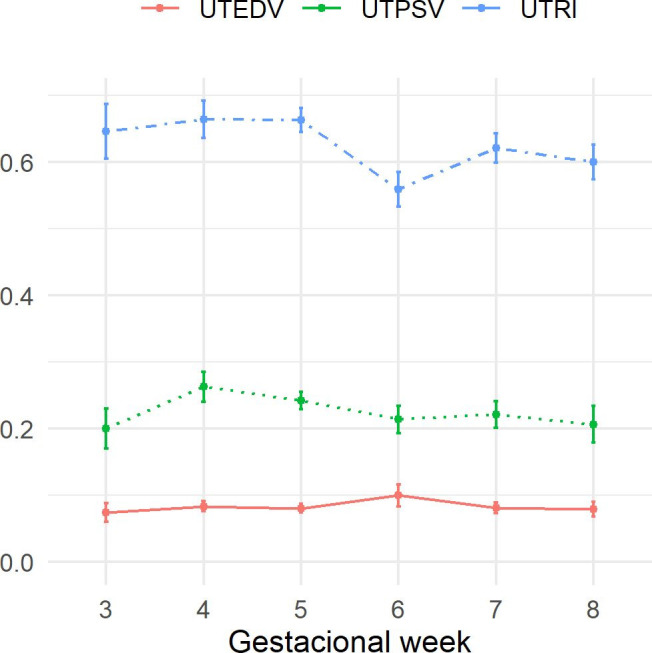
Graph of mean ± standard error of uterine artery’s end diastolic velocity (cm/s), peak systolic velocity (cm/s) and resistance index, of Santa Inês sheep throughout pregnancy. Greater values observed in the last three weeks for EDV and PSV despite (*P*<0.055) and marked reduction in RI in the last gestational week.

End diastolic velocity and the peak systolic velocity of the uterine artery did not present significant variation during pregnancy (*P*>0.055) despite revealing an increase in the last three weeks. The resistance index was higher in the initial weeks, with a marked reduction in the last week of pregnancy (Figure 2).

### Umbilical artery

**Figure 3:**
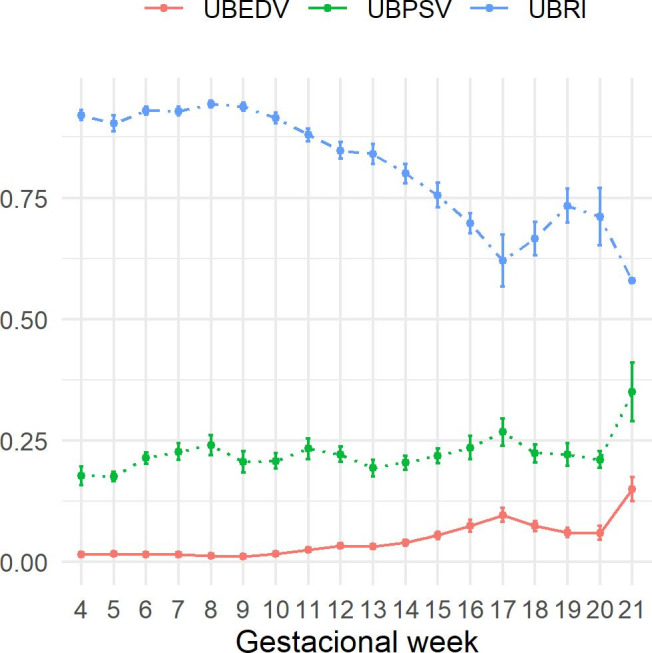
Graph of mean ± standard error of umbilical artery’s end diastolic velocity (cm/s), peak systolic velocity (cm/s) and resistance index, of Santa Inês sheep fetuses throughout pregnancy. It was observed significant increase in EDV (*P*<0.001); no difference for PSV (*P*<0,1) and significant reduction in RI (*P*<0.001).

An increase in the end diastolic velocity was observed with the advance pregnancy (*P*<0.001). For the peak systolic velocity, there was no significant difference (*P*<0.1). The resistance index presented a significant difference (*P*<0.001), with the highest values recorded in the initial weeks, with an evident reduction after the 10^th^ week (Figure 3).

## Discussion

As noticed by Reynolds et al. (1986), changes in blood flow observed in the present study are expected and result from physiological changes in blood vessels related to the gestational process due to the growing nutritional demand of fetuses and placental growth. Changes involve an increase in blood volume and reduction in the resistance index and are a consequence of an increase in the diameter of the vessels (Ousey et al., 2012). High resistance rates would be related to fetal growth retardation in women (Ogueh et al., 2011).

There are few studies on the evaluation of vascular indexes in sheep during pregnancy. Those by Elmetwally et al. (2016) and Beltrame et al. (2017) are two of the most novel. These authors assessed the blood flow in the uterine artery, however, there is a lack of information about other vessels related to pregnancy such as umbilical artery and fetal aorta artery. The knowledge about the blood flow in these vessels can contribute to a more complete understanding about the physiological gestational hemodynamics in sheep, which gives singular importance to this study and its results.

To predict the vitality of lambs by means of fetal eco-biometry, Vannucchi et al. (2019) affirm that there is a relationship between it and lambs’ body weight at birth. They also point the importance of biometry to diagnose normal pregnancies. Our study focused on normal pregnancies and the values of the doppler evaluation determined are related to the birth of healthy lambs, therefore, this could also be a parameter to monitor the correct development of pregnancy.

The adequate blood supply is key to a healthy pregnancy, therefore, the monitoring and determination of vascular indices proposed in our study are of paramount importance. Although there are already previous data on vascular parameters of ewes during pregnancy, more specific data related to the presence of wool can provide more accurate information since it has been described by Junior et al. (2019) that the mean weights of the fetuses were higher (3.688 kg) in shorn ewes when compared to unshorn ewes (3.596 kg) and that the average placental weights also differed between the groups (2.287 kg and 1.923 kg, respectively); showing that the wool factor has an influence on placental development and, consequently, on vascularization and adequate nutrition of the fetuses.

Silva et al. (2018) conclude that their data show that preterm neonates are less adapted to the odds of labor and to overcome the immediate changes of extra-uterine life. The authors did not assess vascularization, but they used clinically healthy ewes, so it is possible to infer that the duration of gestation is as important as a normal vascular development.

For the same reason, it can be assumed that the observations of Korkmaz and Emsen (2016) about birth weight and survival rate of lambs, may be related to more efficient vascular adaptations in crossbred animals. Such adaptations could be anatomical (larger diameter of vessels) or functional (earlier reduction of the RI) that could result in more blood reaching the fetus.

Regarding the uterine artery, no difference was identified for PSV and EDV. There was only a decrease in the resistance index values. Beltrame et al. (2017) report an increase in the peak systolic velocity and the end diastolic velocity and a reduction in the resistance index. The uterine artery was evaluated transrectally until the 8^th^ week of pregnancy and transabdominally from then on. In the period when the route used coincides between the two studies (up to the 8^th^ week) the authors also did not observe a significant difference in the vascular indices.

Elmetwally et al. (2016) used the transrectal route until the 14^th^ gestational week and after that period, the transabdominal route, a methodology similar to that used in this study, and found a decrease in the resistance index and an increase in the peak systolic velocity along of pregnancy. The changes seem to be more marked after the 6^th^ gestational week, and the peak systolic velocity showed a significant increase in the 8^th^ week. These changes are explained by the growth of the fetus and placental development, which increase the need for a larger blood supply (Fowden et al., 2006).

Reduction in the resistance index found in this study is in agreement with the results found by the aforementioned authors and considering the expected physiological changes, it was the only variable that reflected what is expected as a result of the necessary adaptations to the normal development of pregnancy and the fact that the reduction in the index is accentuated in the last week, may indicate that the RI is related to the proximity of delivery.

In the study of the umbilical artery, with the exception of the peak systolic velocity, the changes observed on the end diastolic velocity and in the resistance index are consistent with those observed by Beltrame et al. (2017) and Elmetwally et al. (2016) in the uterine artery and are explained by the increasing need for greater blood supply to the developing fetus.

As reported by Riesen et al. (2002) the increase in end diastolic velocity results in an increase in blood flow and a reduction in the resistance index. What also applies to the umbilical artery according to Serin et al. (2010). These authors also observed a marked reduction in the resistance index after the 85^th^ day of gestation (13^th^ week) in goats. Also observed in this study. They report a second moment of reduction happening on the 130^th^ (19^th^ gestational week). It was only observed in this study in the last week of pregnancy and this reduction may be related to the proximity of delivery, as it seems to occur in bitches according to (Giannico et al., 2015).

In Human Medicine, blood flow in the fetal aorta has been studied, at least, since the 1980’s. It has been reported that the absence of diastolic flow in the fetal aorta is related to the delay in fetal development, and it is related to the prediction of diseases in neonates (Hackett et al., 1987). Blood flow in the fetal aorta was also assessed in women with hypertension and changes in the end diastolic velocity and, in some patients also in the peak systolic velocity, which can be related to this condition (Cameron et al., 1988).

In this study, as pregnancy progressed, an increase in the end diastolic velocity, a reduction in the resistance index and in the peak systolic velocity were observed. Changes in the resistance index and in the end diastolic velocity indicates that blood flow is being facilitated, this may mean that the fetus becomes less dependent on maternal circulation and the fetal aorta assumes increasing importance in the fetal blood circulation.

As reported by Reynolds et al. (2005) there is an increase in placental vascularization during pregnancy in sheep due to increases in both the number and the superficial density of capillaries. The authors also point out that these changes are accompanied by an exponential increase in umbilical and uterine blood flow and an increased placental expression of the endothelial vascular growth factor and its receptor.

The restriction of blood flow in cotyledons, which results in increased resistance, caused changes in the blood flow of the umbilical artery in sheep (Adamson et al., 1990). These authors suggest that there is a high correlation between the blood flow of the umbilical artery and the cotyledons, with the increase in resistance in the cotyledons, causing a reduction in end diastolic velocity, even eliminating this index of the umbilical cord wave.

In this study, an increase in end diastolic velocity was observed in the umbilical artery. As only normal pregnancies were evaluated and this result differs from that found by the aforementioned authors, who induced increased resistance in cotyledons, it is suggested that there is a correlation between blood circulation in these vessels.

Several statistical tests were performed, but no difference was detected between simple, twin and triplet pregnancies. This is because there is a large difference of *n* between groups. Although this fact does not exclude the possibility that there are differences, in this work, it was not possible to detect it.

## Conclusion

The study of blood flow in vessels involved in the gestation of sheep, in addition to the uterine artery, and with weekly frequency is important to acquire a more complete picture of hemodynamic changes resulting from this process. The results obtained are expected to contribute to a broader understanding of the hemodynamic changes resulting from pregnancy.
